# Distinct Effects of Inflammation on Cytochrome P450 Regulation and Drug Metabolism: Lessons from Experimental Models and a Potential Role for Pharmacogenetics

**DOI:** 10.3390/genes11121509

**Published:** 2020-12-16

**Authors:** Laura M. de Jong, Wim Jiskoot, Jesse J. Swen, Martijn L. Manson

**Affiliations:** 1 Division of BioTherapeutics, Leiden Academic Centre for Drug Research (LACDR), Leiden University, 2333 CC Leiden, The Netherlands; l.m.de.jong@lacdr.leidenuniv.nl (L.M.d.J.); w.jiskoot@lacdr.leidenuniv.nl (W.J.); 2 Department of Clinical Pharmacy & Toxicology, Leiden University Medical Center (LUMC), 2333 ZA Leiden, The Netherlands; j.j.swen@lumc.nl; 3 Leiden Network for Personalised Therapeutics, Leiden University Medical Center, 2333 ZA Leiden, The Netherlands

**Keywords:** cytochrome P-450 enzyme system, drug metabolism, hepatocytes, inflammation, inter-individual variability, pharmacogenomics, phenoconversion, phenotype, pregnane X receptor

## Abstract

Personalized medicine strives to optimize drug treatment for the individual patient by taking into account both genetic and non-genetic factors for drug response. Inflammation is one of the non-genetic factors that has been shown to greatly affect the metabolism of drugs—primarily through inhibition of cytochrome P450 (CYP450) drug-metabolizing enzymes—and hence contribute to the mismatch between the genotype predicted drug response and the actual phenotype, a phenomenon called phenoconversion. This review focuses on inflammation-induced drug metabolism alterations. In particular, we discuss the evidence assembled through human in-vitro models on the effect of inflammatory mediators on clinically relevant CYP450 isoform levels and their metabolizing capacity. We also present an overview of the current understanding of the mechanistic pathways via which inflammation in hepatocytes may modulate hepatic functions that are critical for drug metabolism. Furthermore, since large inter-individual variability in response to inflammation is observed in human in-vitro models and clinical studies, we evaluate the potential role of pharmacogenetic variability in the inflammatory signaling cascade and how this can modulate the outcome of inflammation on drug metabolism and response.

## 1. Introduction

The clinical outcome of drug treatments can vary greatly between individuals and even within the same individual. Consequently, certain patients may (suddenly) experience reduced efficacy or exhibit an increased risk for developing adverse events [1]. While part of this variability can be explained by genetic variations in drug-metabolizing enzymes (DMEs)—mainly stemming from the cytochrome P450 (CYP) enzyme family—other non-genetic factors may also greatly contribute to the observed variability in drug response [2].

Inflammation or disease state is shown to have major effects on the metabolism of drugs through downregulation of CYP enzymes and hence contribute to the mismatch between the genotype predicted drug response and the actual phenotype—a phenomenon better known as phenoconversion [3]. However, the impact of inflammation-induced phenoconversion may differ greatly between individual patients and can be dependent on multiple factors. First, the degree of inflammation can significantly influence the extent of CYP suppression [4]. Indeed, signature markers of inflammation are often inversely correlated with drug metabolism [5,6]. Secondly, the type of inflammation or cytokine profile is an important determinant in the effect of inflammation on drug metabolism. Evidently, interleukin-targeting biologics have shown cytokine-specific successes in reversing the repressing effects of inflammatory cytokines towards CYP proteins [7,8]. Thirdly, the extent of inflammation-induced phenoconversion might be dependent on the metabolic pathway of a drug since inflammation is shown to downregulate CYP activities in an isoform-specific manner [9]. Lastly, since drug metabolism is also greatly dependent on genetic variability this might be a fourth factor that alters the extent of inflammation-induced phenoconversion in patients [2,10].

To personalize and optimize drug treatments, a better understanding is needed of how inflammation affects pharmacokinetic behavior and clinical effectiveness of drugs. One major hurdle is that the specific effects of inflammation on pharmacokinetics cannot be easily assessed with in-vivo studies, due to the presence of many interfering covariables (e.g., age, genetic backgrounds, kidney function, co-medication). Therefore, in-vitro liver models may be valuable tools to elucidate the specific effects of inflammation on drug metabolism. Earlier studies with in-vitro models have already demonstrated that various inflammatory mediators associated with inflammation and infection can modulate drug metabolism by reducing the expression of CYPs [2,3,11,12]. However, since the effect of inflammation-induced phenoconversion depends on the degree and type of inflammation as well as the metabolic pathway of the drug, it is necessary to better understand the different effects of various pro-inflammatory mediators and focus on the differential sensitivity between CYP isoforms in response to them.

The aim of this literature review is therefore to (1) summarize and update the available evidence on the effects of inflammatory stimuli on CYP expression levels and activity in human in-vitro liver models, with a specific focus on type of inflammation and metabolic pathway of the drug. (2) Provide an overview of our current understanding of the mechanistic pathways via which inflammation in hepatocytes modulates hepatic functions (e.g., transcription factors, enzymes, nuclear receptors) that are critical for drug metabolism. (3) Define how genetic variation in these defined mechanistic pathways may modulate the effect of inflammation on drug metabolism and drug response.

## 2. Effects of Inflammatory Stimuli on CYP Expression Levels and Activity in Human In-Vitro Liver Models

Experimental laboratory studies have been instrumental for our understanding of how inflammation may modulate drug metabolism in the clinic. Through the use of in-vitro hepatocyte models, researchers have investigated which inflammatory mediators can be held responsible for the observed changes in drug metabolism. These studies primarily emphasize the effects of inflammatory stimuli on either the mRNA expression of the major CYPs responsible for drug metabolism or the actual metabolism of probe substrates for these CYPs. Since DMEs show substantial interspecies differences in terms of metabolizing activity and isoform composition, rodent data may not be useful in extrapolation to the clinic [13]. Therefore, we describe the effects of inflammatory stimuli on CYPs in relevant human in-vitro models, summarized in Table 1.

### 2.1. Interleukin-6 (IL-6)

IL-6 is the chief stimulator cytokine in activation of innate immunity in the liver to contribute to host defense [27]. Owing to its role as the main cytokine in the acute phase response (APR), multiple studies have focused on investigating the effect of IL-6 on CYP levels in-vitro.

#### 2.1.1. Maximal Effect (E_max_) (mRNA)

Numerous investigators have confirmed that IL-6 is a potent downregulator of CYP enzymes in both primary human hepatocytes (PHHs) and in the HepaRG cell line, an immortalized human hepatic cell system that retains PHH characteristics but lacks donor variability. Aitken et al. investigated the effect of inflammatory mediators including IL-6 on mRNA expression of CYP2C9, CYP2C19, and CYP3A4 in PHHs [14]. Treatment with IL-6 downregulated mRNA levels for all isoforms studied, but simultaneously revealed profound differences in the magnitude of downregulation, as the expression of CYP3A4 was markedly more reduced than that of CYP2C9 or CYP2C19. A similar observation was made by Dickmann et al. [19] and Klein et al. [15] in PHHs and by Tanner et al. [23] in the HepaRG cell line, who all reported that IL-6 exerted the strongest downregulation on CYP3A4, whereas the effects of IL-6 on other CYPs, most notably CYP2D6, seemed to be more limited. It should be noted from the work of Klein et al. that IL-6 may also induce expression of CYP2E1 in PHHs, which could be relevant for the metabolism of certain anesthetics [15]. Beyond this exception, IL-6 predominantly reduces CYP expression and thus impairs the biotransformation of a wide range of (pro) drugs that are metabolized through CYP enzymes.

#### 2.1.2. Sensitivity between CYPs (mRNA)

The strength of the Dickmann study is that it examined the effects of IL-6 at different concentrations, allowing determination of the potency (EC_50_) and thus rank ordering the responsiveness of the major CYP enzymes following IL-6 exposure [19]. Through this approach this study was able to demonstrate that the exerted effects of IL-6 in PHH occur at physiological relevant concentrations, as similar concentrations of IL-6 have been detected in the circulation of patients with either chronic or acute inflammation [28,29]. Importantly, these investigations revealed that CYP3A4 was also by potency most sensitive to downregulation by IL-6, as IL-6 downregulated CYP3A4 mRNA with an EC_50_ of 0.0032 ng/mL, whereas a 20- to 500-fold higher concentration of IL-6 was needed for downregulation of other CYPs. A similar difference in CYP sensitivity to IL-6 was observed by Rubin et al. in both PHHs and HepaRG cells [17]. Such differences in sensitivity are potentially important as these data suggest that drugs metabolized by CYP3A4 may be affected already at an earlier state during inflammation than drugs that rely on other CYP enzymes.

#### 2.1.3. Sensitivity between PHH Donors

Another point of attention is the observed interindividual variability in response to IL-6 between donors in a single experimental setup, excluding inconsistencies observed between studies due to model variations or treatment differences [30]. For example, Dickmann et al. reported EC_50_ values for CYP1A2 activity suppression between 0.142 and 4.07 ng/mL (ranging 29-fold) over five donors and a range between 0.0042 and 0.176 ng/mL (ranging 42-fold) for CYP3A4 activity suppression [19]. Evers et al. also reported that CYP3A4 downregulation upon IL-6 stimulation varied largely between donors in one experimental setup, reporting EC_50_ suppression values over approximately a 20-fold range between donors. [30]. The observed different susceptibility to inflammation between donors may be a consequence of both genetic variability and differences in disease state or medical history of the studied donors.

#### 2.1.4. Drug-Metabolizing Activity

Determination of the cytochrome P450 enzymatic activity is important because beyond the described transcriptional effects, posttranscriptional mechanisms may also contribute to the effects of inflammation on drug metabolism [14,31]. As can be observed from Table 1, effects of inflammation have commonly been assessed by measuring metabolite formation of probe substrates of CYP3A4 (midazolam/testosterone), CYP1A2 (phenacetin), CYP2C9 (tolbutamide), CYP2C19 (S-mephenytoin), and CYP2D6 (propafenone/dextromethorfan). Klein et al. showed in PHHs trends for reduced metabolite formation upon IL-6 treatment but statistical power was lacking, presumably because of the heterogeneity of the donors and potential pharmacogenetic variation in CYP450 enzymes as confounding factors [15]. The HepaRG cell line lacks interindividual variability and showed stable CYP expression in the control group over at least 72 h, increasing the reproducibility of the results. In this model, the highest suppression of activity was noted for CYP3A4 as compared to other CYPs, in line with the observed transcriptional downregulation. Tanner et al. showed decreased downregulation of activity for CYP3A4, CYP1A2, and CYP2C19 but not CYP2C9 and CYP2D6 after 24 h [23].

#### 2.1.5. Pathways

IL-6 may exert its effects in hepatocytes via distinct pathways, as the binding of IL-6 to its receptor initiates cellular signaling pathways via three arms, the Janus kinase (JAK)/STAT protein-3 (STAT3) pathway, the mitogen activated protein kinase (MAPK)/extracellular regulated kinase 1 and 2 (ERK1/2) pathway, and the phosphatidylinositol 3-kinase/protein kinase B (PI3K/AKT) pathway [32]. Keller et al. found that, using chemical inhibitors in IL-6 treated PHHs, especially the MAPK/ERK and PI3K/AKT signaling pathways—and not the canonical JAK/STAT pathway—were critical for downregulation of CYP enzymes during inflammation [33]. However, it should be noted that the effect of individual kinase inhibitors was tested in only one individual donor. In contrast, Febvre-James et al. found that treatment with the JAK inhibitor ruxolitinib completely reversed the IL-6-mediated suppression of CYP1A2 and CYP3A4 mRNA levels in both HepaRG cells and PHHs, suggesting a prominent role of the JAK/STAT pathway in CYP downregulation [16]. As such, multiple signaling arms of the IL-6 pathways can be held responsible for the observed downregulation of CYP enzymes.

#### 2.1.6. Long-Term Studies

Implementing long-term investigation on inflammation-induced CYP suppression in-vitro could aid in a better understanding of the chronic inflammation frequently observed in a clinical setting. Long-term investigations on the effect of inflammatory mediators on CYP expression are scarce, especially in PHHs since CYP expression rapidly declines over time in this model [34]. Long et al. investigated the effect of IL-6 on CYP3A4 activity in a 3D microreactor platform with PHH and Kupffer cells [21]. They tested the effect of tocilizumab, an anti-IL-6 receptor antibody, on inflamed hepatocytes and found that coadministration of tocilizumab with IL-6 after initial 4-day IL-6 treatment prevented the CYP3A4 activity decrease across donors. This highlights that the model is capable of capturing physiological adaptation to inflammation, since CYP3A4 desuppression occurred. Tanner et al. collected data on the long-term effects of IL-6 treatment (14 days) in HepaRG cells, which resulted in more pronounced downregulation of P450 expression as compared to short-term treatment [23]. Still, current studies do not address the impact of long-term low concentrations of cytokines as compared to single high-dose treatment, which leaves an open question.

#### 2.1.7. Clinic

Interestingly, the effects of inflammation on drug metabolism in the clinic, most commonly assessed through the IL-6 regulated marker C-reactive protein (CRP), seems to be most reported for CYP3A4 substrates including midazolam, tacrolimus, and/or voriconazole, and less for drugs metabolized through other metabolic pathways. This is in line with data from in-vitro hepatocyte models where IL-6 exerts most profound effects on CYP3A4 [4]. Altogether, these data confirm isoform specific effects of IL-6 and suggest that drugs metabolized via CYP3A4 may be more prone to the effects of inflammation.

### 2.2. Interleukin 1 (IL-1)-Family: Interleukin-1β and Interleukin-18

The IL-1 family compromises a group of 11 proteins that play a role in the initiation and regulation of inflammatory responses, of which IL-1*β* is the most studied member [35].

#### 2.2.1. Maximal Effect (E_max_) (mRNA)

In PHHs, IL-1*β* treatment reduced CYP3A4 mRNA expression with 95%, but it had no effect on CYP2C9 or CYP2C19 mRNA levels [14]. Protein levels of CYP3A4, but also of CYP2C9, were significantly downregulated after 24 h of treatment with IL-1β. Dickmann et al. showed donor-wide suppression (*n* = 5) for CYP3A4/A5, however, IL-1β-mediated suppression of other CYP isoforms (CYP2C9, C19, and 1A2) was not consistently observed among all donors [24]. The observed nonresponse towards IL-1*β* of certain CYP isoforms cannot simply be explained by a lack of effect, since IL-1*β* consistently reduced CYP3A4 expression by >80% in all donors. Alternatively, because the nonresponsive CYP isoforms (CYP2C9 or CYP2C19) differed between donors, nonresponse to IL-1*β* can perhaps be explained by pharmacogenetic variation within these CYP isoforms. IL-18 treatment in HepaRG cells and PHHs did not result in significant downregulation of mRNA levels nor CYP activity [17].

#### 2.2.2. Sensitivity between Models

Interestingly, although Klein et al. showed that the maximal impact of Il-1*β* and IL-6 on the mRNA expression of CYP isoforms was comparable in HepaRG cells, IL-1β showed an approximate 100-fold higher potency than IL-6 in inducing the same downregulation [15]. This described difference in potency might be underlined by the fact that the HepaRG cell line displays morphological heterogeneity, including clusters with nonparenchymal cells which could aggravate or sensitize the response to an inflammatory mediator [36]. For example, IL-1*β* release is associated with activation of the inflammasome in Kupffer cells [37], providing a feed-forward stimulus for production of more inflammatory cytokines which could potentially aggravate cytokine-induced downregulation of CYPs. Indeed, coculturing of Kupffer cells increased responsiveness to IL-1*β* as compared to monocultures of hepatocytes, as evident from an EC_50_ shift from >5 to 0.098 ng/mL for CYP3A4 suppression upon coculturing, an effect not seen with IL-6 treatment [20,22]. Since IL-18 is also reported to mediate its effect through Kupffer cells [38], this can explain the lack of effect on CYPs in HepaRG or PHHs cell models described by Rubin et al. Thus, inclusion of nonparenchymal cells in model systems might increase the responsiveness to IL-1*β* and IL-18 and hence better reflect the potential effect these inflammatory cytokines may have in an intact human liver.

#### 2.2.3. Sensitivity between PHH Donors

Looking at the suppression of activity of CYP3A4 and CYP1A2 in PHHs upon IL-1*β* treatment, again large interdonor variation is evident [24]. Dickmann et al. found an average EC_50_ value for two donors of 0.450 ng/mL (three donors showed no response) regarding CYP1A2 activity. For CYP3A4, EC_50_ values for activity ranged from 0.005 to 1.06 ng/mL over five donors.

#### 2.2.4. Pathways

The effects of IL-1β are presumed to be mediated via activation of the nuclear factor kappa B (NF-κB) pathway [39]. Importantly, IL-1*β* may also rapidly (within 2–4 h) induce IL-6 expression, which raises the possibility that part of the observed actions of IL-1β are actually mediated by IL-6 [40]. Interestingly, a recent study by Febvre-James et al. found that the IL-1*β* repression of CYP enzymes could not be reversed by the JAK inhibitor ruxolitinib, confirming that IL-1*β* and IL-6 induce distinct pathways upon inflammation and may complement one another in altering drug metabolism [16].

### 2.3. Tumor Necrosis Factor α (TNF-α)

TNF-*α* is another main cytokine involved in inducing the acute phase response in the liver during inflammation. Hepatocytes express the tumor necrosis factor receptor 1 (TNFR1) that upon binding by TNF-α results in the activation of the major NF-κB pathway and the MAPK/ERK pathway [41]. Aitken et al. found that TNF-*α* treatment induced CYP3A4 mRNA downregulation but not protein downregulation after 24 h [14]. They saw no effect on CYP2C9 and CYP2C19 mRNA levels upon TNF-α treatment, but interestingly the CYP2C9 protein levels were reduced by >95% after 24 h treatment, pointing to a mismatch between the effects on mRNA and protein expression levels. This suggests that post-transcriptional mechanisms, i.e., protein degradation or regulation by miRNAs, are involved in downregulation of CYP protein levels by TNF-α. In line, Dallas et al. reported no effects of TNF-*α* on CYP2C19 and CYP2C9 mRNA levels, but found significantly downregulated CYP2C19 and CYP2C9 activity [18]. Klein et al. found that TNF-α treatment resulted in similar downregulation of CYP gene expression in HepaRG cells as observed with IL-6 treatment, presuming that part of the effect of TNF-α is mediated via nonparenchymal cells [15]. After 72 h of exposure to TNF-*α*, all P450 activities were reduced by more than 80%.

### 2.4. Pathogen Associated Molecular Patterns (PAMPs)

PAMPs, such as lipopolysaccharide (LPS), are microbial molecules that can signal immune cells to destroy intruding pathogens associated with infection [42]. Upon LPS recognition, the toll like receptor 4 (TLR4) signaling pathway ultimately activates NF-kB. The study by Aitken et al. found LPS to be the most efficacious inflammatory stimulus in downregulating mRNA levels of CYP3A4, and CYP3A4 protein levels were decreased by about 75% of control 24 h after treatment [14]. Whereas LPS treatment did not influence mRNA levels of CYP2C9 or CYP2C19, CYP2C9 protein levels were reduced by 80% after 24 h of treatment, again indicating a mismatch between mRNA and protein levels. This is in accordance with data presented by Rubin et al. in HepaRG cells and PHHs, where LPS downregulated CYP3A4 and CYP1A2 mRNA levels in both models [17]. LPS showed comparable potency in downregulating CYP3A4 compared to IL-6, but was much less potent in downregulating CYP1A2 levels.

### 2.5. Other Cytokines: Transforming Growth Factor β (TGF-β), Interferon γ (IFN-γ), Interleukin-22 (IL-22), Interleukin-23 (IL-23), and Interleukin-2 (IL-2)

The effect of other pro-inflammatory mediators beyond IL-6, IL-1*β*, TNF-*α*, and PAMPs has also been studied in in-vitro hepatocyte models. TGF-*β*, an inflammatory mediator linked to fibrosis, caused significant downregulation of CYP3A4, CYP2C9, and CYP2C19 mRNA levels and subsequent protein levels (only shown for CYP3A4 and CYP2C9) [14]. Interestingly, only TGF-*β* and IL-6 downregulated CYP2C9 mRNA, but protein expression levels of CYP2C9 were strongly downregulated by all inflammatory stimuli tested. IFN-γ, a mediator that is associated with the immune response to viral infections, only reduced mRNA levels of CYP3A4 in PHHs. Conversely, IL-22, a pro-inflammatory mediator found in different auto-immune disorders, was found to repress mRNA levels of CYP1A2, CYP3A4, and CYP2C9 in PHHs and HepaRG cells [25]. Studies investigating the effect of IL-2 on CYP3A4, 1A2, 2C9, 2C19, and 2D6 expression found no suppression of mRNA levels upon treatment in PHHs [18,20]. Interestingly, when culturing the hepatocytes together with Kupffer cells, a concentration-dependent decrease (50–70%) of CYP3A4 activity was observed with IL-2 at 72 h, suggesting that Kupffer cells are essential for the suppressive effect of IL-2 [20]. IL-12 and IL-23, pro-inflammatory mediators associated with inflammatory autoimmune responses, did not impact CYP3A4 levels [26] and a coculture model did not change this [22]. The effect of other cytokines on CYP expression and activity is yet to be determined.

### 2.6. Summary

The in-vitro data summarized here suggests that direct treatment with inflammatory stimuli can suppress DMEs stemming from the CYP1, CYP2, and CYP3 family. This suppressive effect is most convincingly demonstrated for IL-6, IL-1*β*, TNF-*α*, and LPS. CYP3A4 seems to be most susceptible to cytokine-induced downregulation in human in-vitro hepatocyte models, whereas CYP2D6 seems to be the least sensitive. The enzyme expression of CYP1A2, CYP2C9, and CYP2C19 was also sensitive to the effects of inflammatory mediators, though higher concentrations of cytokines were in general required to downregulate these enzymes and the response was not always conserved among all studied donors. Interestingly, model-dependent responses were observed which could be reliant on the presence of nonparenchymal cells. The effect of inflammatory mediators should therefore be divided into direct effects on hepatocytes and indirect effects through inflammatory signaling in nonparenchymal cells.

Importantly, interdonor variation in response to inflammation within the same experimental setup was observed. Translating these findings to the clinic, the consequences of inflammation-induced phenoconversion for drug treatments may differ therefore greatly between individuals and between the metabolic CYP pathways via which drugs are metabolized.

## 3. Mechanistic Pathways via Which Inflammation Modulates Hepatic Functions That Are Critical for Drug Metabolism

The above described findings from in-vitro models show that the sensitivity to inflammation may differ between CYP isoforms and inflammatory stimuli. This implies that distinct mechanisms are involved in the downregulation of CYP enzyme expression and activity.

**Figure 1:**
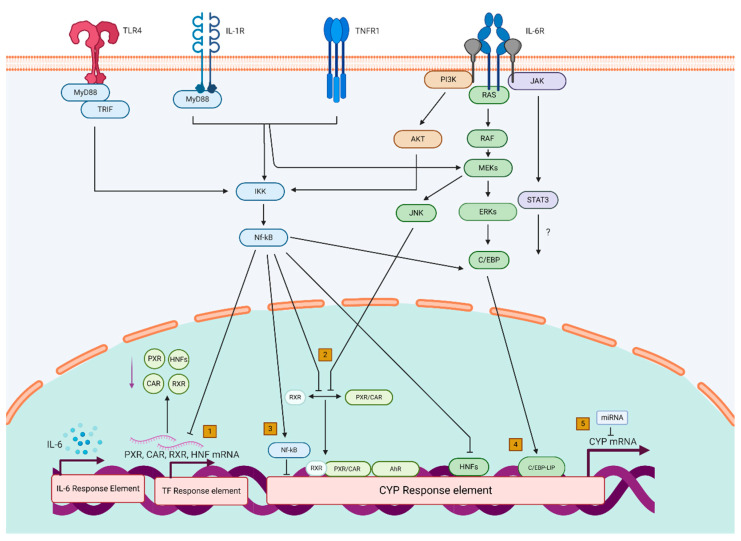
Mechanistic insights into the effects of inflammation on CYP expression and activity. Transcriptional repression of important CYP enzymes during inflammation may proceed through (1) transcriptional downregulation of nuclear receptors and other transcription factors, (2) interference with dimerization/translocation of nuclear transcription factors, (3) altered liver-enriched C/EBP signaling, (4) direct regulation by NF-κB, or (5) posttranscriptional mechanisms.

Mechanistically, regulation of hepatic CYP levels and interactions with CYP gene regulators is complicated and includes a wide variety of ligand-activated transcription factors and mediators. Cytokine-mediated alteration of gene transcription is thought to be the main regulatory mechanism accountable for changing CYP450 activity upon inflammation. It is essential to note that no single common pathway is recognized for all the CYP enzymes and underlying mechanisms are cytokine-specific. Here we describe, summarized in Figure 1, how repression of important CYP enzymes during inflammation may proceed through (1) transcriptional downregulation of transcription factors, (2) interference with dimerization/translocation of (nuclear) transcription factors, (3) altered liver-enriched C/EBP signaling, (4) direct regulation by NF-κB, or (5) post-transcriptional mechanisms.

### 3.1. Transcriptional Downregulation of Transcription Factors

Transcription factors involved in the regulation of CYP mRNA levels, including the nuclear receptors pregnane X receptor (PXR), the constitutive androstane receptor (CAR), their dimerization partner retinoid X receptor (RXR), the aryl hydrocarbon receptor (AhR), as well as human nuclear factors (HNFs) are held responsible for the observed downregulation of DMEs upon inflammation. It is important to distinguish between the role of nuclear transcription factors in the constitutive expression of CYP enzymes versus drug- or inflammation-mediated expression. Here we will focus on the nuclear hormone receptor mechanisms likely to be involved in inflammation-altered CYP expression.

#### 3.1.1. Downregulation of Nuclear Receptors

The PXR (gene: *NR1I2*) and the CAR (gene: *NR1I3*) are members of the nuclear receptor superfamily highly expressed in the enterohepatic system of mammals [43]. These ligand activated transcription factors have been identified as key transcriptional regulators of the cytochrome P450 xenobiotic-metabolizing enzymes, mostly for the CYP2C9, CYP2C19, CYP3A4, and CYP3A5 enzyme expression [44]. Upon binding with the RXR, the heterodimer nuclear receptor-RXR complex binds to responsive elements present in the 5′-flanking regions of target genes, usually resulting in an upregulation of gene expression aimed at increased metabolism of drugs. Studies have indeed shown that PXR and CAR increase transcription of the human CYP3A4/5, CYP2C9, CYP2C19, and CYP1A2 genes upon drug treatment [45,46,47].

One mechanism by which inflammation changes gene transcription of major DMEs is through repression of the nuclear receptor PXR and CAR. A vast body of evidence shows that inflammation represses PXR levels, leading to downregulation of important CYP enzymes. Pascussi et al. pioneered in showing that IL-6 downregulates PXR mRNA in PHH and inhibits the rifampicine-induced induction of CYP3A4 [48]. Upon LPS treatment in HepG2 cells, the mRNA and protein levels of PXR are reduced [49]. Mechanistically, a decrease in PXR expression within the nucleus was observed, leading to reduced transactivation of the CYP3A4 promotor and subsequent inhibited transcriptional activity of CYP3A4. Additionally, LPS treatment in mice led to functional repression of PXR’s dimerization partner RXR [50]. Yang et al. showed that inhibition of a CYP3A4 promotor reporter after IL-6 treatment in human hepatocytes was greater in the presence of PXR than after its knockdown, suggesting a role for PXR in IL-6-facilitated suppression of CYP3A4 [51]. Knockdown of PXR in human hepatocytes reversed the IL-6-induced CYP3A downregulation. Furthermore, the authors suggest that downregulation of PXR by inflammatory stimuli is causative for decreased transcription of CYP3A4: a continuous decrease in PXR levels was observed already after 1.5 h of treatment, whereas a significant decrease in CYP3A4 mRNA levels occurred only after 3 h. A likely scenario is that the suppressive effect of inflammation on PXR expression is mediated through NF-κB activation, since Zhou X et al. showed that NF-kB directly interacts with a functional binding site in the PXR promotor to suppress its transcriptional expression [52]. Transcriptional downregulation of CAR upon inflammatory stimuli has also been reported. A study by Assenat et al. investigated the negative regulation of CAR via pro-inflammatory cytokines IL-1*β* and LPS in human hepatocytes [53]. IL-1*β* treatment reduced mRNA levels of CYP2B6, CYP2C9, and CYP3A4 through NF-κB p65 activation. This p65 subunit of the NF-κB complex interfered with the distal glucocorticoid response element present in the CAR promotor, leading to repressed transcription of CAR. In contrast, the AhR is not substantially affected by IL-6 treatment [15,23]. As such, it appears that the response to inflammation is substantial for PXR and CAR and their dimerization partner RXR, but not for AhR.

Still, some debate remains about the role of nuclear receptors in the downregulation of CYP enzymes during inflammation, mostly stemming from conflicting rodent vs. human studies. In the rodent field, a study by Beigneux et al. suggested that downregulation of PXR and CAR was causative for CYP450 downregulation [50], whereas other experiments suggest that downregulation of important P450 enzymes does not necessitate the nuclear receptor PXR. As an example, Richardson et al. found that downregulation of multiple CYP mRNAs was similar in LPS-treated control and PXR-null mice, suggesting a PXR independent mechanism [54]. For the human situation, transcription factors responsible for the homeostasis of CYPs are evidently downregulated through inflammation. However, up to what extent downregulation of these transcription factors can actually be held responsible for the inflammation driven changes in expression of DMEs and drug metabolism itself remains to be further investigated.

#### 3.1.2. Downregulation of Hepatocyte Nuclear Factors

Hepatocyte nuclear factors (HNFs), including HNF-1α and HNF-4*α*, form another important family of transcription factors. They can modulate CYP expression in the liver through DNA-binding interactions in CYP promotors or via modulation of PXR and CAR expression [55,56,57,58,59]. Despite their well-documented role in CYP homeostasis, the contribution of HNFs for the inflammation-induced changes in CYP expression remain, however, scarcely investigated.

The binding activities of HNF-1*α* and HNF-4*α* to DNA were quickly reduced in rat livers treated with LPS in parallel with downregulated hepatic CYP mRNA levels [60]. In HepG2 cells, treatment with IL-6 and IL-1*β* resulted in a 10% decrease of HNF-4*α* activity as a result of an altered phosphorylation status [61]. Acute and prolonged treatment with IL-6 reduced mRNA levels of HNF-4a in HepaRG cells, but this effect was not seen for HNF-1a and the changes shrink into insignificance compared to the observed downregulation of, e.g., PXR [23]. In contrast, Klein et al. found that mRNA levels of the HNF-4*α* were downregulated (≈40%) by IL-6 only at the early time point of 8 h and seemed to have normalized after 24 h [15]. However, a direct link between the fast transcriptional suppression of P450 genes and the reduced mRNA levels/activity of HNFs is still lacking, questioning a prominent role of transcriptional HNF downregulation as a factor in IL-6-induced DME suppression.

### 3.2. Interference with Dimerization/Nuclear Translocation of (Nuclear) Transcription Factors

Impairment of the activity of important transcription factors could, in addition to the above described transcriptional repression of transcription factors, also contribute to repression of CYPs during inflammation. Tanner et al. questioned whether transcriptional downregulation of PXR and CAR mRNA levels itself can fully explain the observed downregulation of CYP enzymes [23]. They suggested that the transactivation potential of PXR and CAR might be simultaneously influenced by inflammation. They found a clear correlation between downregulated PXR and CYP mRNA levels after short-term treatment with IL-6. However, the reduction in PXR expression following prolonged treatment (14-days) with IL-6 was very modest compared to the downregulation observed for the CYP enzymes. As such, downregulation of nuclear receptor target genes (e.g., CYPs) during inflammation could be a consequence of decreased availability of PXR itself, or an impairment of the translocation/activity of the receptor.

The existence for such interactions between inflammation and hepatic transcription factors (PXR, CAR, and AhR) have been suggested for both the NF-κB pathway and pathways related to IL-6 signaling. A hypothesized mechanism for this is interference of NF-κB with the dimerization of PXR to RXR and subsequent binding to DNA, thereby inhibiting the activity of PXR. The inhibited transcriptional activity of PXR leads to downregulation of DMEs in HepG2 cells [62]. As NF-κB interferes with the binding of RXR to PXR, this mechanism of repression by NF-κB may also hold true for more nuclear receptor-controlled systems where RXR is the dimerization partner (e.g., CAR), but no experimental evidence exists that can yet support this. AhR-regulated CYP1A2 is likely not regulated by this mechanism. Studies in mouse hepatoma cells have shown that interactions between the P65 subunit of NF-κB and AhR may result in the formation of an inactive complex, with possible consequences for the translocation to the nucleus [63]. In addition, NF-κB has been shown to inhibit transcriptional activity of AhR by reducing histone acetylation of promotors of CYP enzymes (e.g., CYP1A2), thereby altering the accessibility of the DNA for nuclear transcription factors [64]. Thus, activation of the NF-κB pathway may modulate the activity of nuclear transcription factors through changes in dimerization, translocation, or chromatin remodeling.

Kinases involved in the IL-6 signaling pathway can also alter the protein status and translocation of nuclear receptors. Cell signaling protein kinases such as Jun-N-terminal kinase (JNK) and protein kinase C (PKC) can repress the activity of the nuclear receptors PXR and CAR, thereby altering their function and impact on downstream transcriptional CYP activity [65,66,67]. One hypothesized mechanism is that kinases can alter the phosphorylation status of these nuclear receptor proteins. IL-1β treatment induces JNK expression which can phosphorylate RXR, leading to reduced nuclear binding activity and subsequently inhibited RXR-dependent hepatic gene expression [68]. Additionally, LPS-induced downregulation of P450 genes was attenuated upon treatment with a specific JNK inhibitor in a primary mouse hepatocyte model [69]. Thus, JNK can play a role in inflammation-mediated downregulation of nuclear receptors with RXR as partner. This was backed up by findings from Ghose et al., who showed that an increase in JNK signaling is associated with higher export of RXR out of the nucleus upon low-dose LPS treatment, leading to less RXR-mediated hepatic gene expression [70]. Additionally, ERK signaling has been proven to impair nuclear translocation of CAR in a mouse primary hepatocyte model [71]. Altogether, these findings indicate that kinases play an important role in the regulation of nuclear receptors and their dimerization with RXR, thereby offering a general mechanism for the suppression of genes regulated by nuclear receptors during inflammation. How other important inflammatory cell-signaling components in the IL-6 pathway, such as STAT3, mechanistically regulate CYP repression remains to be investigated.

### 3.3. Direct Regulation by NF-κB

NF-κB can precisely control the expression of CYP1A1, CYP2B1, CYP2C11, CYP2D5, CYP2E1, and CYP3A7 via interaction with the promotors of these genes, leading to downregulation in most cases [72]. For example, Iber et al. reported that the CYP2C11 promotor region contains a low-affinity binding place for NF-κB and mutations in the 3′-end or 5′-end in this NF-κB response element reduced the binding affinity for NF-κB and subsequently suppressed CYP2C11 transcription by IL-1 or LPS in rat hepatocytes [73]. However, experimental evidence for this hypothesis has only been obtained in animal models. Although there is high conservation of CYP enzymes amongst species, the extent and catalytic activity between species differs, highlighting that caution should be taken in extrapolation of results to a human situation [13].

### 3.4. Altered Liver-Enriched C/EBP Signaling

The expression and DNA binding activity of the transcription factor C/EBPβ is severely enhanced during the acute phase liver response through activation of the NF-κB pathway [74]. One mechanism that is hypothesized to contribute to CYP repression upon IL-6 stimulation is altered balance between two isoforms of the transcription factor C/EBP-β: the liver-enriched transcriptional activating protein (LAP) and the liver-enriched transcriptional inhibitory protein (LIP). The LIP isoform is a shortened variant of C/EBPβ deficient of transactivation activity. Jover et al. found upregulation of the C/EBPβ-LIP protein isoform in HepG2 cells treated with IL-6 [75]. They demonstrated that LIP antagonized transactivation of CYP3A4 by the functional LAP isoform. This altered LAP:LIP ratio correlated with a downregulation of CYP3A4 enzyme levels. Martinez et al. showed a novel enhancer site located in the CYP3A4 gene where the LAP isoform can bind and initiate transcription, whereas the antagonizing action of the truncated LIP isoform on LAP resulted in CYP3A4 gene repression, confirming that the LAP:LIP ratio is of importance in regulation of constitutive expression of CYP3A4 [76]. A C/EBPβ-based mechanism was also found to be involved in transcriptional repression of CYP2A6 [77]. It is yet to be determined whether this mechanism can also explain repression of other CYPs upon IL-6 stimulation in a human model.

### 3.5. Posttranscriptional Mechanisms (miRNA)

The mechanisms behind downregulation of DMEs upon inflammation, as described above, remain an area of intense study. Increasing attention is being given to the potential post-transcriptional mechanisms that could regulate P450 enzymes in inflammation as well. MicroRNAs (miRNAs) can influence the translation and stability of cellular mRNAs at their 3′-UTR side, offering a broad mechanism for gene expression regulation [78]. Previous research has already shown that miRNA activity regulates phase I and II metabolizing enzymes and transcriptional factors through posttranscriptional modification [31,79,80].

A recent study by Kugler et al. questions whether the previously observed mechanisms are sufficient to explain the huge downregulation of DMEs observed upon inflammation and investigated the possible role of miRNA in this process [81]. They performed transfections with five inflammation-associated miRNAs in HepaRG cells and looked at the CYP mRNA levels and activity. They found miRNA-dependent downregulation of several CYP mRNA and expression levels after 96 h, where CYP2C19 and CYP3A4 were amongst the top downregulated genes. Thus, miRNAs might be an extra factor in downregulating drug metabolizing capacity during inflammation. Potentially, this could also explain the sometimes observed mismatch between CYP mRNA levels and CYP protein levels after inflammatory stimuli, as was the case for CYP2C9 in the study from Aitken et al. [14]. Since the 3′-UTR region of CYP2C9 can directly be regulated by miR-130b, this could explain the downregulation of CYP2C9 enzyme expression. As such, miRNA regulation could (in part) be responsible for the effects of inflammatory mediators on protein levels in the absence of preceding downregulation of mRNA.

Other post-transcriptional mechanisms, such as the role of nitric oxide in the cytokine-mediated regulation of CYPs were excellently reviewed by Morgan et al. [82].

### 3.6. Concluding Remark

Concluding from previous sections, we hypothesize that the variation in sensitivity of different CYP enzymes for inflammation stems from the distinct mechanisms that regulate them. It seems like PXR- and CAR-regulated CYP enzymes (3A4/5, 2C9, 2C19) are more sensitive to inflammation, whereas the AhR regulated isoform CYP1A2 is less sensitive. CYP2D6 shows to be least sensitive to inflammation, which might be due to the fact that it is not inducible by nuclear receptors and therefore not sensitive to inflammation-induced alterations of the levels of PXR, CAR, and AhR that regulate the expression of other CYPs [83,84,85]. Most interestingly, deduction of CYP specific inflammatory mechanisms of downregulation can shed light on the distinct sensitivities towards inflammation.

## 4. Pharmacogenetic Variation in Inflammatory Pathways and the Effect on Drug Pharmacokinetics

The available data from in-vitro experiments with PHHs on drug metabolism have indicated that the response to inflammation or its inflammatory mediators may differ substantially between donors under controlled experimental conditions [19,24,30]. This raises the question whether the observed distinct response to inflammation between persons is also observed in the clinic. Clinical studies by van Wanrooy et al. and Vet et al. have shown that the metabolism of voriconazole and midazolam at similar concentrations of CRP and corrected for other known confounding factors may still vary considerably between patients [5,6]. These findings from both in-vitro models and clinical studies suggests the existence of interindividual variability with regards to the effects of inflammation on drug metabolism. This distinct response towards inflammation between subjects may in part be caused by genetic variability in the described pathways via which inflammation modulates the activity of DMEs.

By presenting examples from the available literature we illustrate how genetic variability within the different elements presented in Figure 1 can modulate the outcomes of the effect of inflammation on drug metabolism and consequently may contribute to the observed interindividual variability in the effect of inflammation.

### 4.1. Genetic Variation: Inflammatory Mediators

It is well established that genetic variability within inflammatory mediators (e.g., cytokines) can predispose individuals to an altered susceptibility to immune-related disease [86]. For this reason, it is plausible that polymorphisms in cytokine genes could shape the immune response that affects drug metabolism. One prominent example relates to the rs1946518 (-607C/A) variant within the promoter of IL-18 and its effects on the metabolism of the immunosuppressive tacrolimus. Xing et al. and Zhang et al. demonstrated that Han-Chinese patients carrying the AA genotype (19–29% of the patients) exhibited lower concentration/dose (C/D) ratios of tacrolimus within the first month after lung or kidney transplantation than patients with an AC or CC genotype [87,88]. Interestingly, this relationship for the rs1946518 variant was exclusively shown for patients expressing CYP3A5*1 and functionally linked to lower expression of IL-18 mRNA in the liver. These results imply that the rs1946518 variant reduced the IL-18 driven inflammation in the liver, which prevents the inflammation-induced downregulation of CYP3A5 and consequently reduces the impact of inflammation on drug metabolism in these patients. Importantly, rs1946518 did not modulate C/D ratios in liver transplant patients who were already treated for 1 year with tacrolimus [89]. These results suggest that the variant only affects drug metabolism shortly after transplantation when the immune/inflammatory responses are highest. Altogether, this example illustrates that genetic variability within inflammatory mediators has the potential to modulate the effects of inflammation on drug metabolism.

### 4.2. Genetic Variation: Inflammatory Receptors

As described above, toll-like receptor (TLR) activation by pathogen-associated molecules may downregulate CYP3A4 expression and modulate drug metabolism. However, TLR activation may also be triggered by endogenous molecules (e.g., DNA) that are released during ischemia-reperfusion injury that develops during organ transplantations [90]. Therefore, it has been postulated that genetic variability in TLRs may alter the effect of inflammation and its consequences for drug metabolism. Ou et al. showed that liver transplant patients with the TLR9-rs352139 AA genotype exhibited lower C/D tacrolimus levels than carriers of the AG/GG genotype [91]. Subsequent cellular experiments provided functional support for these observations and demonstrated that the TLR9-rs352139 variant impaired TLR9 expression and consequently reduced NF-κB activation. TLR9-rs352139 AA genotype carriers were thus protected from the effects of ischemia-reperfusion-induced inflammation, which resulted in conservation of their metabolic capacity. The opposite effect was observed for carriers of the TLR4-rs1927907-GG phenotype who exhibited higher tacrolimus C/D ratios than AA/AG carriers, indicating that these patients were more susceptible to the effects of inflammation on their drug-metabolizing capacity [91,92]. These studies illustrate that genetic variants in receptors can be important modulators of inflammation, which may be particularly relevant for receptors (e.g., IL6R or IL-1R) that are directly involved in the downregulation of CYP enzymes, but this remains to be investigated.

### 4.3. Genetic Variation: Inflammatory Transcription Factors (NF-κB)

Genetic variability within NF-κB is of great interest given its essential role in inflammatory signaling [93]. One common polymorphism in the *NFKB1* gene is the promotor -94 ATTG insertion/deletion mutation (rs28362491), with a minor allele frequency of 0.43. Deletion of the ATTG alleles is shown to reduce synthesis of the NF-κB p50 subunit [94]. Zhang et al. showed that patients with the *NFKB1* -94 ATTG ins/ins genotype had higher CYP3A4-metabolized dose-adjusted cyclosporine trough concentrations than patients with the -94 ATTG del/del genotype [95]. The impact of the same polymorphism in *NFKB1* on the pharmacokinetics of lovastatin, a cholesterol-lowering drug mainly metabolized by CYP3A4, was also investigated [96]. In accordance, the area under the plasma concentration–time curve (AUC) of the metabolite lovastatin lactone was twofold higher in subjects with two copies of the *NFKB1*-94 ATTG ins/ins mutation and the plasma clearance was lower as compared to the *NFKB1*-94del/del genotype. The *NFKB1*-94del/del mutation may thus impair inflammatory signaling and hence attenuate the inflammation-induced downregulation of CYP3A4. Consequently, patients with the *NFKB1*-94del/del genotype may perceive milder consequences of inflammation on drug metabolism than people lacking this variant. Since NF-κB is a downstream effector molecule of several inflammatory cytokines, genetic variability has the potential to simultaneously alter the actions of multiple inflammatory mediators on CYP gene expression. The potential impact of genetic variability within NF-κB or within the genes of NF-κB adaptor proteins on the effects of inflammation on drug metabolism is therefore predicted to be greater than genetic variability in the receptors or the mediators themselves.

### 4.4. Genetic Variation: Nuclear Receptors (PXR, CAR)

The nuclear receptors PXR and CAR are, as highlighted earlier, important for the transcriptional regulation of CYP450 enzymes. Pharmacogenetic variations within the genes encoding PXR (*NR1I2*) or CAR (*NR1I3*) has therefore been thoroughly investigated in relation to their effects on pharmacokinetics and efficacy of drug treatments, as reviewed comprehensively by Mbatchi et al. [97]. However, the influence of genetic variants within *NR1I2* or *NR1I3* has primarily been linked to homeostatic regulation of CYP expression in the absence of inflammation. Until now, it remains therefore largely unclear which genetic polymorphisms in *NR1I2* or *NR1I3* might be candidates for modulating the effects of inflammation on drug metabolism.

Since PXR is regulated by NF-κB, either through direct transcriptional repression or via interference with RXR-PXR binding, we hypothesize that polymorphisms within *NR1I2* that present themselves in or near NF-κB binding sites might influence the impact of inflammation on drug metabolism [98]. For this reason we used the computational databases “gene transcription regulation database” (GTRD) and “Alggen PROMO database” for identification of polymorphisms in *NR1I2* that would be susceptible to the effects of inflammation [99,100,101]. Using information on confirmed NF-κB binding sites by chromatin immunoprecipitation-sequencing (CHIP-seq) or predicted NF-κB binding spots, we were able to identify four common variants (minor-allele frequency > 0.01) in *NR1I2* that are located in or near NF-κB binding spots, as summarized in Table 2. Importantly, the variant *NR1I2*-rs3814055 that has initially been linked to a NF-κB binding site was not confirmed by this approach, which is in accordance with observations from Dring et al. who also did not find evidence for a NF-κB binding site positioned at the rs2814055 location [102].

The effects on drug metabolism of these four genetic variants in the NF-kB binding spots in *NR1I2* are sparsely reported in the literature. This may suggest that these variants contribute less than other common SNPs within *NR1I2* (e.g., rs3814055, rs2472677) to the variability of drug metabolism in the absence of inflammation. However, some data is available from studies conducted in cancer patients. Inflammatory reactions are frequently observed in cancer patients and a common cause of phenoconversion [3,103].

Interestingly, in a cohort of 109 patients with colon cancer, the “inflammatory” variant *NR1I2* rs10934498 (G > A) was identified, from a panel of *NR1I2* variants, as one of the main determinants of Irinotecan pharmacokinetics [104]. Irinotecan is a prodrug that is converted into its active metabolite SN-38 and subsequently detoxified into SN-38G. Patient with the rs10934498 AA genotype exhibited reduced SN-38 AUC levels and increased metabolic ratios of SN-38G compared to AG or GG carriers, which indicates that the metabolism of Irinotecan is more conserved in patients with the rs10934498 AA genotype. Based on our observation that rs10934498 is located in an NF-κB binding site, we hypothesize that PXR may no longer be downregulated by inflammation in patients carrying the rs10934498 AA genotype, resulting in a conserved drug-metabolizing activity compared to patients lacking this variant.

Altogether, the computational identification of common “inflammatory” variants within *NR1I2* suggest that genetic variability may modulate PXR-dependent outcomes of inflammatory signaling. However, further (functional) studies are needed to elucidate the impact of these *NR1I2* polymorphisms on drug metabolism in the context of inflammation.

### 4.5. Genetic Variation: Cytochrome P450 Enzymes

Ultimately, the output of the inflammatory signaling cascade regulates CYP expression and subsequent drug metabolic capacity. Even though it is well established that genetic polymorphisms in CYP enzymes contribute to the interindividual variability in pharmacokinetics [2], it remains uncertain how and to what extent CYP polymorphisms may modulate the impact of inflammation on drug metabolism. Some studies hint towards a genotype-dependent effect of inflammation-induced phenoconversion, as summarized by Klomp et al. [105]. CYP2C19 is highly polymorphic and shown to be affected by inflammation. For example, in a study of 34 patients with an invasive fungal infection receiving voriconazole, it was shown that the effect of inflammation was modulated by the CYP2C19 genotype: the metabolic ratio of voriconazole and its metabolite was more decreased by inflammation in CYP2C19 ultrarapid metabolizers compared to CYP2C19 intermediate metabolizers [106]. Similarly, Ohnishi et al. aimed to investigate the consequences of inflammation for different CYP2C19 genotypes by examining the metabolic ratios of omeprazole and its metabolite in hepatitis C virus (HCV)-positive patients and healthy volunteers [107]. The shift in metabolic ratio between healthy patients and HCV-positive patients was largest for genotype-predicted normal metabolizers (21.1-fold change), followed by intermediate metabolizers (12.4-fold change) and least evident for poor metabolizers. Although these examples only illustrate the effects of inflammation on CYP2C19 mediated drug metabolism, and other CYP isoforms remain to be investigated, they clearly indicate that inflammation-induced changes in CYP450-mediated drug metabolism are affected by an individual’s CYP metabolizer genotype.

## 5. Conclusions

Concluding, data from in-vitro models have been instrumental to elucidate that CYP isoforms show distinct susceptibility to downregulation by inflammatory mediators wherein CYP3A4 seems to be most affected by inflammation, supporting clinical observations on CYP3A4 drug substrates. Additionally, the pattern of downregulation of CYP isoforms was dependent on the inflammatory stimulus. Interestingly, interindividual variability in response to inflammation is observed in both in-vitro models and clinical studies. Genetic variability in the described pathways via which inflammation modulates the expression and activity of DMEs might in part explain the distinct response towards inflammation between subjects, but this remains to be further investigated. Ultimately, a better understanding of inflammation-induced phenoconversion may aid in optimizing treatment for the individual patient.

## Figures and Tables

**Table 1:** Regulation of Clinically Important Drug-Metabolizing Cytochrome P450 Enzymes by Pro-Inflammatory Stimuli in Relevant Human in-Vitro Models.

Stimulus	Model	Effect on CYP mRNA Expression				Effect on Drug Metabolism					Ref.
		Duration	Studied Concentration	Maximal Effect (%)	Potency (EC_50_ ng/mL)	Duration	Drug	Metabolite	Potency (EC_50_ ng/mL)	Maximal Effect (%)	
IL-6	PHH	24 h	10 ng/mL	CYP3A4 (↓95%)							[14]
				CYP2C9 (↓35%)							
				CYP2C19 (↓40%)							
	PHH	24 h	10 ng/mL	CYP3A4 (↓85%)		72 h	atorvastatin	o-OH-atorvastatin		NS	[15]
				CYP1A2 (↓76%)			phenacetin	acetaminophen		NS	
				CYP2C9 (↓65%)			tolbutamide	OH-tolbutamide		NS	
				CYP2C19 (↓41%)			S-mephenytoin	4′-OH-mephentoin		NS	
				CYP2D6 (↓41%)							
				CYP2E1 (↑402%)							
	PHH	24 h	10 ng/mL	CYP3A4 (↓97%)							[16]
				CYP1A2 (↓96%)							
	PHH	48 h	0.0006–50 ng/mL	CYP3A4 (↓90%)	0.454						[17]
				CYP1A2 (↓80%)	5.49						
	PHH *	48 h	10 ng/mL	CYP3A4 (↓98%)		72 h	testosterone	6*β*-hydroxytestosterone		↓76%	[18]
				CYP1A2 (↓27%)			phenacetin	acetaminophen		↓22%	
				CYP2C19 (↓72%)			S-mephenytoin	4′-OH-mephentoin		↓65%	
				CYP2C9 (↓63%)			tolbutamide	OH-tolbutamide		↓35%	
				CYP2D6 (↑240%)			dextromethorphan	dextrorphan		↓39%	
	PHH	72 h	0.005–50 ng/mL	CYP3A4 (↓95%)	0.0032	72 h	testosterone	6*β*-hydroxytestosterone	0.073	↓70%	[19]
				CYP3A5 (↓95%)	0.051						
				CYP1A2 (↓85%)	0.271		phenacetin	acetaminophen	1.25	↓90%	
				CYP2C19 (↓80%)	0.071						
				CYP2C9 (↓90%)	0.121						
				CYP2D6 (↓70%)	0.151						
	PHH/PHH:KC (10:4)		2–200 ng/mL	CYP3A4 (?)		72 h	testosterone	6*β*-hydroxytestosterone		↓90%	[20]
	PHH:KC *		0.001–10 ng/mL	CYP3A4 (?)		96 h	luminogenic P450-Glo™ substrate	proluciferin substrate	0.463	↓80%	[21]
	PHH:KC (10:4)	96 h	0.00625–5 ng/mL	CYP3A4 (↓95%)		96 h	luminogenic P450-Glo™ substrate	proluciferin substrate	0.252	↓ > 95%	[22]
	HepaRG	24 h	10 ng/mL	CYP3A4 (↓95%)		24 h	midazolam	1′-hydroxymidazolam		decreased	[23]
				CYP3A5 (↓90%)							
				CYP1A2 (↓80%)			phenacetin	acetaminophen		decreased	
				CYP2C9 (↓85%)			tolbutamide	OH-tolbutamide		NS	
				CYP2C19 (↓85%)			S-mephenytoin	4′-OH-mephentoin		decreased	
				CYP2D6 (NS)			propafenone	5-hydroxypropafenone		NS	
				CYP2E1 (NS)							
	HepaRG	24 h	10 ng/mL	CYP3A4 (↓93%)		72 h	atorvastatin	o-OH-atorvastatin		↓ > 80%	[15]
				CYP3A5 (↓89%)							
				CYP1A2 (↓90%)			phenacetin	acetaminophen		↓ > 60%	
				CYP2C9 (↓83%)			tolbutamide	OH-tolbutamide		↓ > 60%	
				CYP2C19 (↓83%)			S-mephenytoin	4′-OH-mephentoin		↓ > 60%	
				CYP2E1 (NS)							
	HepaRG	48 h	0.123–30 ng/mL	CYP3A4 (↓99%)	<0.123	72 h	midazolam	1′-hydroxymidazolam	2.89	↓60%	[17]
				CYP1A2 (↓90%)	0.452		phenacetin	acetaminophen	8.96	↓65%	
	HepaRG	48 h	10 ng/mL	CYP3A4 (↓ > 95%)		4 h	midazolam	1′-hydroxymidazolam		↓61%	[16]
				CYP1A2 (↓ > 95%)			phenacetin	acetaminophen		↓68%	
	HepaRG	336 h (14 days)	10 ng/mL	CYP3A4 (NS)		336 h (14 days)	midazolam	1′-hydroxymidazolam		decreased	[23]
				CYP3A5 (↓80%)							
				CYP1A2 (↓95%)			phenacetin	acetaminophen		decreased	
				CYP2C9 (NS)			tolbutamide	OH-tolbutamide		NS	
				CYP2C19 (↓90%)			S-mephenytoin	4′-OH-mephentoin		NS	
				CYP2D6 (NS)							
				CYP2E1 (NS)							
IL-1*β*	PHH	24 h	5 ng/mL	CYP3A4 (↓95%)							[14]
				CYP2C9 (NS)							
				CYP2C19 (NS)							
	PHH	72 h	0.0001–10 ng/mL	CYP3A4 (↓95%)	0.294	72 h	testosterone	6*β*-hydroxytestosterone	0.416	↓90%	[24]
				CYP3A5 (↓62%)	0.347						
				CYP1A2 (↓73%)	0.531 ^#^		phenacetin	acetaminophen	0.45	↓65%	
				CYP2C9 (↓79%)	0.229 ^#^						
				CYP2C19 (↓58%)	0.153 ^#^						
				CYP2D6 (↓75%)	0.945 ^#^						
	PHH/ PHH:KC (10:4)		0.2–200 ng/mL	CYP3A4 (?)		72 h	Testosterone	6*β*-hydroxytestosterone		↓85%	[20]
	PHH:KC (10:4)	96 h	0.00625–5 ng/mL	CYP3A4 (↓ > 95%)		96 h	luminogenic P450-Glo™ substrate	proluciferin substrate	0.098	↓ > 95%	[22]
	HepaRG	24 h	5 ng/mL	CYP3A4 (↓97%)		72 h	atorvastatin	o-OH-atorvastatin		↓ > 80%	[15]
				CYP3A5 (↓91%)							
				CYP1A2 (↓93%)			phenacetin	acetaminophen		↓ > 80%	
				CYP2C9 (↓90%)			tolbutamide	OH-tolbutamide		↓ > 80%	
				CYP2C19 (↓93%)			S-mephenytoin	4′-OH-mephentoin		↓ > 80%	
				CYP2E1 (↓75%)							
	HepaRG	24 h	1 ng/mL	CYP3A4 (↓98%)							[16]
				CYP1A2 (↓99%)							
IL-18	PHH	48 h	1.95–500 ng/mL	CYP3A4 (NS)		72 h	midazolam	1′-hydroxymidazolam		NS	[17]
				CYP1A2 (NS)			phenacetin	acetaminophen		NS	
	HepaRG	48 h	2.06–600 ng/mL	CYP3A4 (NS)		72 h	midazolam	1′-hydroxymidazolam		NS	[17]
				CYP1A2 (NS)			phenacetin	acetaminophen		NS	
TNF-*α*	PHH	24 h	10 ng/mL	CYP3A4 (↓80%)							[14]
				CYP2C9 (NS)							
				CYP2C19 (NS)							
	PHH *	48 h	10 ng/mL	CYP3A4 (↓87%)		72 h	testosterone	6*β*-hydroxytestosterone		↓70%	[18]
				CYP1A2 (↓45%)			phenacetin	acetaminophen		↓72%	
				CYP2C19 (NS)			S-mephenytoin	4′-OH-mephentoin		↓82%	
				CYP2C9 (NS)			tolbutamide	OH-tolbutamide		↓17%	
				CYP2D6 (↓40%)			dextromethorphan	dextrorphan		↓42%	
	HepaRG	24 h	10 ng/mL	CYP3A4 (↓90%)		72 h	atorvastatin	o-OH-atorvastatin		↓ > 80%	[15]
				CYP3A5 (↓79%)							
				CYP1A2 (↓87%)			phenacetin	acetaminophen		↓ > 80%	
				CYP2C19 (↓64%)			S-mephenytoin	4′-OH-mephentoin		↓ > 80%	
				CYP2C9 (↓62%)			tolbutamide	OH-tolbutamide		↓ > 80%	
				CYP2E1 (↓54%)							
TGF-*β*	PHH	24 h	10 ng/mL	CYP3A4 (↓75%)							[14]
				CYP2C9 (↓50%)							
				CYP2C19 (↓50%)							
IFN-y	PHH	24 h	10 ng/mL	CYP3A4 (↓75%)							[14]
				CYP2C9 (NS)							
				CYP2C19 (NS)							
IL-22	PHH	48 h	10 ng/mL	CYP3A4 (↓70%)							[25]
				CYP1A2 (↓45%)							
				CYP2C9 (↓50%)							
	HepaRG	24 h	10 ng/mL	CYP3A4 (↓75%)	1.7	48 h	midazolam	1′-hydroxymidazolam		↓50%	[25]
				CYP1A2 (↓60%)			phenacetin	acetaminophen		↓50%	
				CYP2C9 (↓50%)							
IL-2	PHH *		2–200 ng/mL	CYP3A4 (?)		72 h	testosterone	6*β*-hydroxytestosterone		NS	[20]
	PHH *	48 h	10 ng/mL	CYP3A4 (NS)		72 h	testosterone	6*β*-hydroxytestosterone		NS	[18]
				CYP1A2 (NS)			phenacetin	acetaminophen		NS	
				CYP2C19 (NS)			S-mephenytoin	4′-OH-mephentoin		↓21%	
				CYP2C9 (NS)			tolbutamide	OH-tolbutamide		NS	
				CYP2D6 (↑150%)			dextromethorphan	dextrorphan		↓22%	
	PHH:KC (10:4) *		200 ng/mL	CYP3A4 (?)		72 h	testosterone	6*β*-hydroxytestosterone		↓70%	[20]
	PHH:KC (10:4)		200 ng/mL	CYP3A4 (?)		96 h	luminogenic P450-Glo™ substrate	proluciferin substrate		NS	[22]
IL-12	PHH	48 h	10 ng/mL	CYP3A4 (NS)		48 h	testosterone	6*β*-hydroxytestosterone		NS	[26]
				CYP2C19 (NS)			S-mephenytoin	4′-OH-mephentoin			
				CYP2C9 (NS)			tolbutamide	OH-tolbutamide			
IL-23	PHH	48 h	10 ng/mL	CYP3A4 (NS)		48 h	testosterone	6*β*-hydroxytestosterone		NS	[26]
				CYP2C19 (NS)			S-mephenytoin	4′-OH-mephentoin			
				CYP2C9 (NS)			tolbutamide	OH-tolbutamide			
	PHH:KC (10:4)		200 ng/mL	CYP3A4 (?)		96 h	luminogenic P450-Glo™ substrate	proluciferin substrate		NS	[22]
LPS	PHH	24 h	10 µg/ml	CYP3A4 (↓95%)							[14]
				CYP2C9 (NS)							
				CYP2C19 (NS)							
	HepaRG	48 h	1.37–333 ng/mL	CYP3A4 (↓95%)	<1.37	72 h	midazolam	1′-hydroxymidazolam	7.85	↓60%	[17]

NS = not significant. * = effect of inflammatory mediator on CYP expression and activity was investigated after treatment with a standard CYP-inducer. ^#^ = excluding non-responders. ↑ = increased CYP expression. ↓ = decreased CYP expression. ? = effect of inflammatory mediator on CYP mRNA expression was not determined.

**Table 2:** SNPs in *NR1I2* Located in A Predicted or Confirmed NF-κB Binding Site ^1^.

SNP	Variation	Location	Allele Frequency	In Binding Site (Proximity) of:	Distance to Binding Site (bp)	Binding Spot Predicted in:
rs10934498	G > A, C, T	intron	G = 0.5024	NFκB1-p105 subunit	0	GTRD
			A = 0.4976			
rs1403526	A > C, G	Intron	A = 0.64900	RelA-p65 subunit	0	Alggen PROMO
			G = 0.35100			
rs12721602	G > A	5 -UTR	G = 0.98303	RelA-p65 subunit	13	Alggen PROMO
			A = 0.01697			
rs1054191	G > A, C	3′-UTR	G = 0.87745	NF-κB, NF-κB1 p105	17	Alggen PROMO
			A = 0.12255			

^1^ To cover relevant NF-kB binding sites, we took into consideration the human NF-κB p105 subunit, the NF-κB p100 subunit, and the RelA-p65 subunit binding sites. An arbitrary threshold of 25 base pairs from confirmed or predicted NF-κB binding spot was set to identify relevant *NR1I2* SNPs [98]. Matching score to consensus sequence was set at 85% for the Alggen PROMO database. For the GTRD, CHIP-seq derived data was collected from the meta clusters data set. Allele frequencies were obtained from the GnomeAD or 1000Genomes database.
